# Influence of Working Temperature on The Formation of Electrospun Polymer Nanofibers

**DOI:** 10.1186/s11671-016-1824-8

**Published:** 2017-01-19

**Authors:** Guang-Zhi Yang, Hai-Peng Li, Jun-He Yang, Jia Wan, Deng-Guang Yu

**Affiliations:** School of Materials Science & Engineering, University of Shanghai for Science and Technology, 516 Jungong Road, Yangpu District, Shanghai, 200093 People’s Republic of China

**Keywords:** Electrospinning, Nanofiber, Temperature, Polyacrylonitrile, Polyvinylpyrrolidone

## Abstract

Temperature is an important parameter during electrospinning, and virtually, all solution electrospinning processes are conducted at ambient temperature. Nanofiber diameters presumably decrease with the elevation of working fluid temperature. The present study investigated the influence of temperature variations on the formation of polymeric nanofibers during single-fluid electrospinning. The surface tension and viscosity of the fluid decreased with increasing working temperature, which led to the formation of high-quality nanofibers. However, the increase in temperature accelerated the evaporation of the solvent and thus terminated the drawing processes prematurely. A balance can be found between the positive and negative influences of temperature elevation. With polyacrylonitrile (PAN, with *N*,*N*-dimethylacetamide as the solvent) and polyvinylpyrrolidone (PVP, with ethanol as the solvent) as the polymeric models, relationships between the working temperature (*T*, K) and nanofiber diameter (*D*, nm) were established, with *D* = 12598.6 − 72.9*T* + 0.11*T* ^2^ (*R* = 0.9988) for PAN fibers and *D* = 107003.4 − 682.4*T* + 1.1*T* ^2^ (*R* = 0.9997) for PVP nanofibers. Given the fact that numerous polymers are sensitive to temperature and numerous functional ingredients exhibit temperature-dependent solubility, the present work serves as a valuable reference for creating novel functional nanoproducts by using the elevated temperature electrospinning process.

## Background

Electrostatic energy has gradually increased its share from other types of energies (such as mechanical energy, acoustic energy, and thermal energy) in creating nanoproducts through a top-down manner. Popular examples of such nanoproducts are electrospun nanofibers and electrosprayed nanoparticles [1–3]. In these electrohydrodynamic methods, electrostatic energy performs a dominant function in generation; however, other energies, such as thermal, radiant, and mechanical energies, can be combined into the process for an effective production [4–6].

Currently, the development of electrospinning focuses on two directions. One is the large-scale production of electrospun nanofibers for commercial products through edge, multiple-needle, needle-less, and slit electrospinning [7–9]. However, few studies tackled cost reduction and experimental optimization. A recent publication has demonstrated that the reasonable utilization of spinneret material (polypropylene) can save electric energy and improve the aligned effect of electrospun nanofibers [10]. This paper demonstrates a concept that proposes a substantial development space for optimizing experimental or production conditions to create high-quality nanofibers in an economical manner. Among these conditions, such as applied voltage, fluid flow rate, fiber-collecting distance, environmental humidity, and temperature, working fluid temperature has received the least attention in terms of influence on the formation of electrospun polymer nanofibers from solution electrospinning, although reports regarding melt electrospinning can be found in publications [11].

Another development direction for electrospinning is to generate novel types of nanostructures and nanofibers. These nanostructures include the popular core–sheath fibers, tri-layer nanofibers, Janus nanofibers, and the complicated nanostructures from a combination of core–sheath and Janus [12–15]. However, only slightly over 100 polymers can be electrospun into nanofibers under ambient temperature, thus considerably limiting the potential applications of multiple-fluid electrospinning in creating novel nanostructures and functional nanoproducts. A reasonable selection of temperature of the working fluids can be a useful tool for nanofabrication through electrospinning processes. First, some semicrystalline polymers (such as polyethylene and polypropylene) are dissolved in solvents only at an elevated temperature. Elevated temperature electrospinning creates new types of polymer nanofibers [4]. Second, elevated temperature logically decreases the viscosity of polymer solutions but exerts minimal influence on physical entanglements, which particularly benefit the formation of electrospun nanofibers from polymer species with ultra-high molecular weights [16]. Third, numerous functional nanofibers are created by adding a guest active ingredient into a host filament-forming polymer matrix, whereas numerous ingredients, such as numerous poorly water-soluble drugs, exhibit temperature-dependent solubility [17]. Thus, elevated temperature electrospinning has the potential to expand the capability of electrospinning to generate new functional nanofibers and nanostructures.

Although temperature is a key parameter in electrospinning, the related reports are extremely limited, which is possibly correlated with the simple implementation of electrospinning under ambient conditions. The key in running elevated temperature electrospinning lies in heating and maintaining the working fluid at a constant temperature different from ambient conditions. Steven et al. [4] utilized a ceramic infrared emitter to manipulate solution temperature up to 110 °C during electrospinning. They declared that infrared flux on the polyethylene solution from the emitter can be precisely controlled by both the variable output controller and the distance between the emitter and the glass syringe. Wang et al. [16] reported a jacket-type heat exchanger that was exploited to control the temperature of solutions containing polyacrylonitrile (PAN) in dimethylformamide up to 88.7 °C. A circulation of heated silicone oil by a pumping system connected to an oil bath was utilized to adjust the working temperature. Considering the applications of an electric heating film and a temperature regulator, Yu et al. developed an auxiliary heating system to maintain a constant temperature of working fluids for preparing medicated nanofibers [17, 18] and drug-loaded composite microparticles [19]. Desai and Kit [20] conducted elevated temperature electrospinning to prepare beadless composite nanofibers consisting of chitosan and polyacrylamide. The working temperature of 70 °C was maintained through the circulation of hot air around the syringe and needle. Kin et al. [21] prepared cellulose nanofibers from its solutions in a mixture of *N*-methylmorpholine oxide and water through elevated temperature electrospinning but provided no detailed information on the heating unit. De Vrieze et al. [22] investigated the effects of temperature and humidity on electrospun cellulose acetate nanofibers. The working temperature was adjusted using a polymethylmethacrylate chamber to house the electrospinning system, and the whole setup was placed in a temperature-controlled room, with variations only from 273 to 303 K. These approaches (including infrared radiation, direct heating, indirect heating through flowing air/oil/water, or even storage in a constant temperature room) can be considered when implementing elevated temperature electrospinning over a range of working temperature.

The above-mentioned publications successfully demonstrated the usefulness about the combined utilization of thermal energy and electrostatic energy in creating polymeric nanoproducts with an elevated temperature of the working solutions. However, none of these works systematically investigated the influence of temperature on fiber formation. In the present work, polyacrylonitrile (PAN) and polyvinylpyrrolidone (PVP) were utilized as the model filament-forming polymers. A synthetic and semicrystalline organic polymer resin with the linear formula (C_3_H_3_N)_n_, PAN is a versatile polymer used to produce a large variety of products, including ultrafiltration membranes, hollow fibers for reverse osmosis, and fibers for textiles. This polymer is used as the chemical precursor in 90% of high-quality carbon fiber production and is also extensively used in electrospinning; PAN nanofibers are good precursors for preparing carbon nanotubes (CNTs) [23, 24]. PVP is a water-soluble polymer made from the monomer *N*-vinylpyrrolidone. This polymer is soluble in water and other polar solvents. PVP is used as binders in pharmaceutical tablets and as additives in batteries, ceramics, fiberglass, inks, and inkjet paper; this polymer is also used in the production of membranes, such as dialysis and water purification filters, as well as in the solubility enhancement of poorly water-soluble drugs [25, 26]. PAN and PVP are preferred not only because of their extremely broad applications in a wide variety of fields but also because of their special electrospinnability. PAN possesses spinnability in the aprotic solvent *N*,*N*-dimethylacetamide (DMAc), which displays a high boiling point of 166 °C. PVP features good spinnability in the typical protic solvent ethanol, with a boiling point of 78.4 °C. The two working solutions can represent almost all types of polymer solutions exploited for electrospinning in creating the corresponding nanofibers.

## Methods

### Materials

PAN powders (MW = 80,000) were obtained from Shangyu Baisheng Chemical Technology Co., Ltd. (Shaoxing, China). PVP K90 (MW = 360,000) was purchased from BASF Shanghai Co., Ltd. (Shanghai, China). *N*,*N*-Dimethylacetamide (DMAc) and anhydrous ethanol were provided by Shanghai Chemical Reagent Co., Ltd. (Shanghai, China). All chemicals used were of analytical grade.

### Working Fluids and Electrospinning

Two electrospinnable solutions were prepared to implement elevated temperature electrospinning. One solution contains PAN in DMAc with a concentration of 15% (*w*/*v*). To ensure a homogeneous working fluid, the PAN solution was agitated over 12 h at 80 °C and then was cooled to the ambient temperature. The other solution was PVP K90 in anhydrous ethanol with a concentration of 9% (*w*/*v*) and was prepared under ambient conditions.

A homemade electrospinning system was employed in the preparation processes. The system consisted of a voltage source (ZGF 60 kV/2 mA, Wuhan Huatian Electrical Co., Ltd., Wuhan, China), a pump (KDS100, Cole-Parmer®, Vernon Hills, IL, USA), a spinneret (a stainless steel capillary with an inner hole diameter of 0.32 mm, 23G, O6Cr19Ni10, GB24511, China), a collector (a cardboard wrapped with aluminum foil), and an accessory for heating and manipulating the working fluid temperature. The detailed parameters for creating PAN and PVP nanofibers are included in Table 1.

**Table 1:** Spinning parameters for preparing PAN and PVP nanofibers

No.	Working fluid^a^		Temperature (°C)	Other conditions^b^	
	Polymer	Solvent		Applied voltage	Flow rate
F1	PAN (Con. 15%)	DMAc (BP:166.0)	20 ± 1	14 kV	1 mL/h
F2			40 ± 1		
F3			60 ± 2		
F4			80 ± 2		
F5	PVP K90 (Con. 9%)	Ethanol (BP:78.5)	20 ± 1	10 kV	2 mL/h
F6			30 ± 1		
F7			40 ± 1		
F8			50 ± 1		
F9			60 ± 2		

^a^Con. means concentration in *w*/*v*, and BP means boiling point in °C

^b^The fiber-collected distance was fixed at 15 cm

### Characterizations

The morphology of electrospun fibers was assessed through field-emission scanning electron microscopy (FESEM; Quanta FEG450, FEI Corporation, Hillsboro, OR, USA). Prior to examination, samples were sputter-coated with platinum to prevent charging during FESEM imaging. ImageJ software (National Institute of Heath, Bethesda, MD, USA) was utilized to measure the fiber diameter from SEM micrographs. For each sample, nanofiber size was measured at over 100 points.

The surface tensions and viscosities of the PVP solution were measured as a function of working temperature. The former was carried out with a BZY-1 Surface Tension Tensiometer (Shanghai Hengping Instrument and Meter Factory, Shanghai, China). The latter was conducted using a NDJ-279 rotary viscometer (Machinery and Electronic Factory of Tongji University, Shanghai, China). An HZBZ-08 Automatic saturated vapor pressure measuring instrument (Shanghai Xu-Ji Electric Co., Ltd., Shanghai, China) was exploited to measure the saturated vapor of anhydrous ethanol at different temperatures (20.0–78.0 °C) by using a static method [27].

## Results and Discussion

### Implementation of Elevated Temperature Electrospinning

**Fig. 1:**
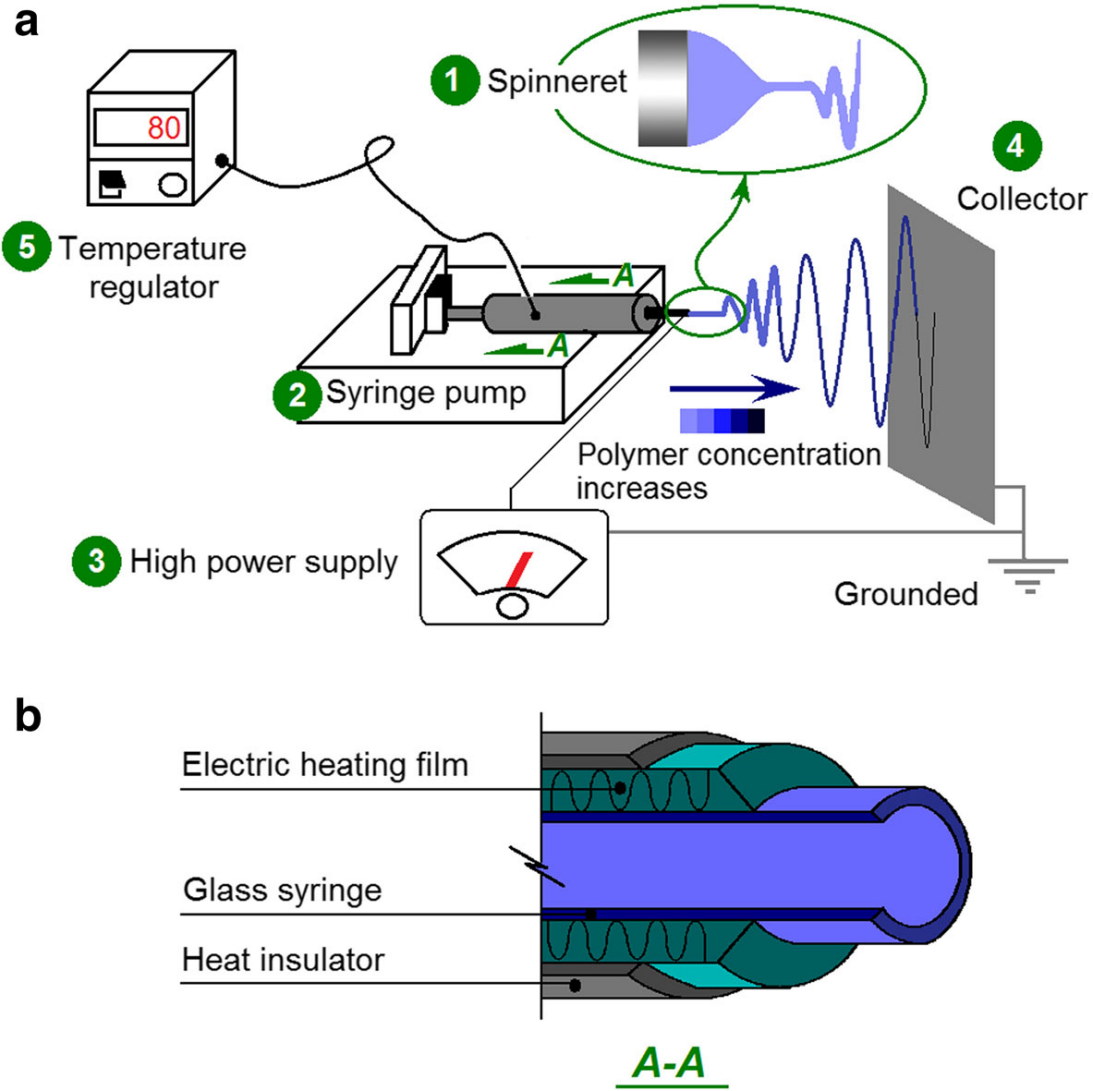
Schematic of elevated temperature electrospinning. **a** Five components of the electrospinning system: (*1*) spinneret, (*2*) syringe pump, (*3*) high power supply, (*4*) collector, and (*5*) temperature regulator and the accessory. **b** Diagrammatic cross-section of the heating and temperature maintenance accessory

A typical electrospinning system consists of four components (Fig. 1), namely, the spinneret (to direct the working fluid to the electric field), the syringe pump (to drive and meter the working fluid), the high-voltage generator (to provide the electrostatic energy), and the fiber collector. Based on the typical system, the elevated temperature electrospinning system can be simply built by introducing a heating and temperature maintenance accessory. Both direct heating/radiation and indirect heat transfer through hot air/oil can be utilized to maintain the working fluid at a fixed temperature that is higher than the ambient condition.

In the present work, the inner structure of the auxiliary apparatus is shown in Fig. 1b. The ambient temperature was maintained at 20 °C under room air conditioners. Other temperature levels exceeding this value were achieved by the heating film. Before applying high voltages to commence electrospinning, the working fluids were first equilibrated for half an hour at the pre-determined temperature. The auxiliary temperature-controlled accessory possessed good temperature-regulated accuracy with a fluctuation of ±2 °C.

**Fig. 2:**
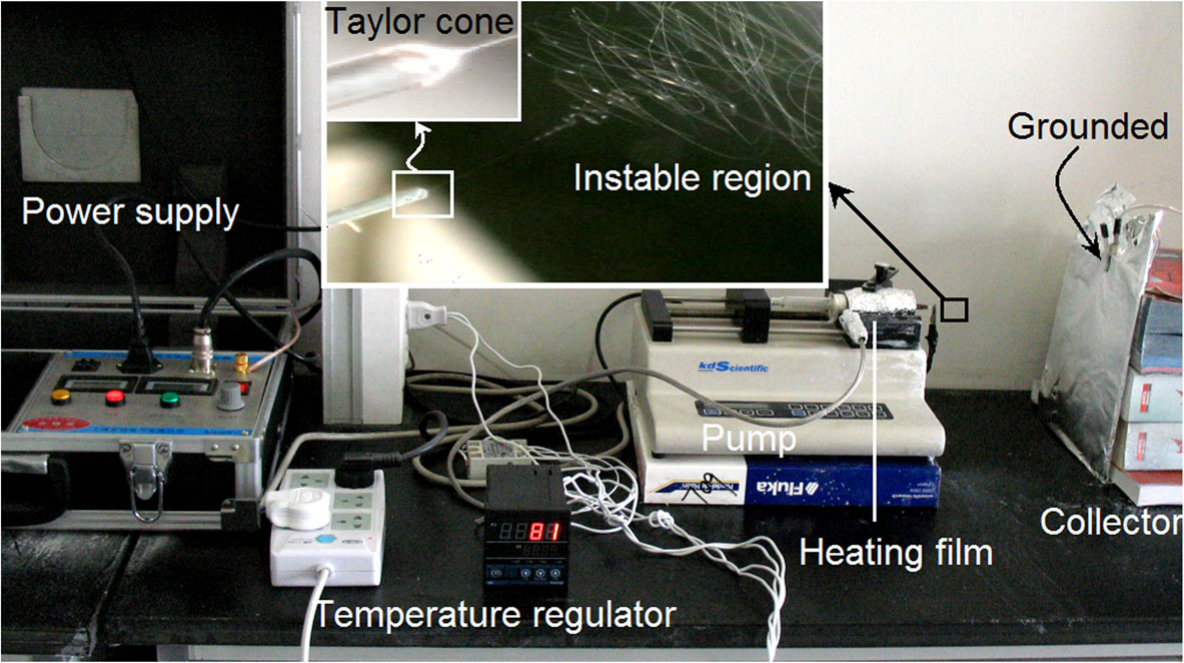
Implementation of elevated temperature electrospinning. The *insets* show the observations of Taylor cone and instable region. Electrospinning conditions: an applied voltage of 14 kV, a flow rate of 1 mL/h, and a temperature of 80 °C of the 15% (*w*/*v*) PAN solution in DMAc

All electrospinning processes of PAN solutions were implemented smoothly and continuously under the selected operative conditions, including a series of working temperatures of 20, 40, 60, and 80 °C. A digital image of the running processes is exhibited in Fig. 2, which was taken when PAN nanofibers were fabricated at 80 °C. The upper-middle insets are images of a typical Taylor cone and the instable region comprising enlarged circles and loops. During the electrospinning processes, the PAN fluid jets flew to the fiber collector in a direction that was slightly inclining to the upper-right because the fiber collector was grounded using an alligator clip on its top, which was higher than the spinneret. This phenomenon suggests that the gravity force acting on the fluid jets was extremely small. Compared with the electrical drawing and the attractive forces between the two electrodes, the influence of gravity could be ignored.

### Influence of Temperature on The Formation of PAN Nanofibers

**Fig. 3:**
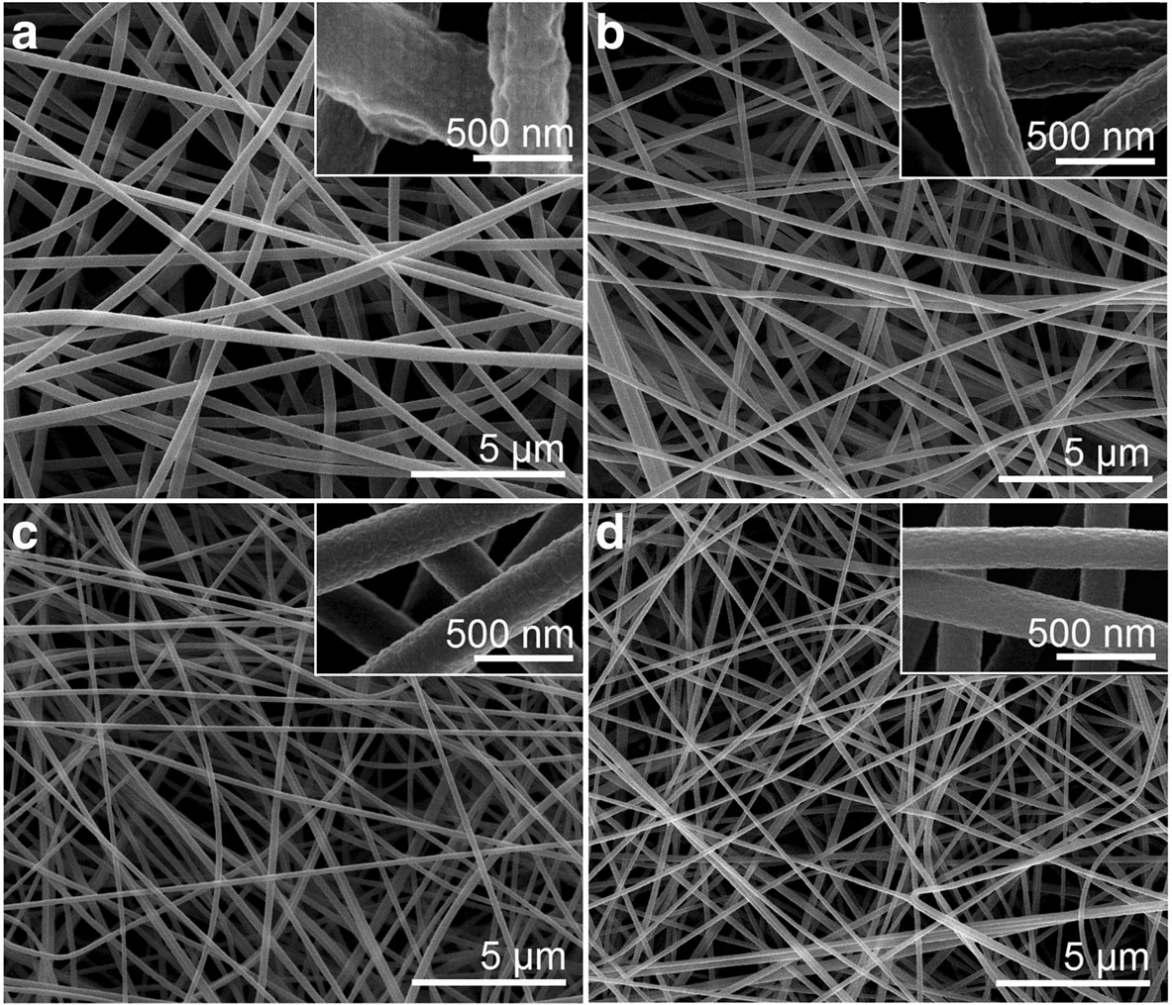
SEM images of the prepared PAN nanofibers under different working temperatures. **a** F1, ambient temperature (20 °C), **b** F2, 40 °C, **c** F3, 60 °C, and **d** F4, 80 °C. The *upper-right insets* show enlarged images of the corresponding PAN fibers

Four types of PAN nanofibers prepared under different working temperatures are shown in Fig. 3, and their detailed surface morphology are presented in the corresponding upper-right insets. All four types of PAN nanofibers possessed fine linear morphology without any discerned beads-on-a-string or spindles-on-a-string phenomenon. All PAN nanofibers were distributed uniformly. These results suggest that the elevation of working temperature exerted no negative influence on the electrospinnability of the PAN working solutions. However, the enlarged images in the upper-right insets depict significantly different surface smoothness of the PAN nanofibers. As shown in the insets of Fig. 3a–d, higher working temperature corresponded to smoother surface of the as-prepared PAN nanofibers.

**Fig. 4:**
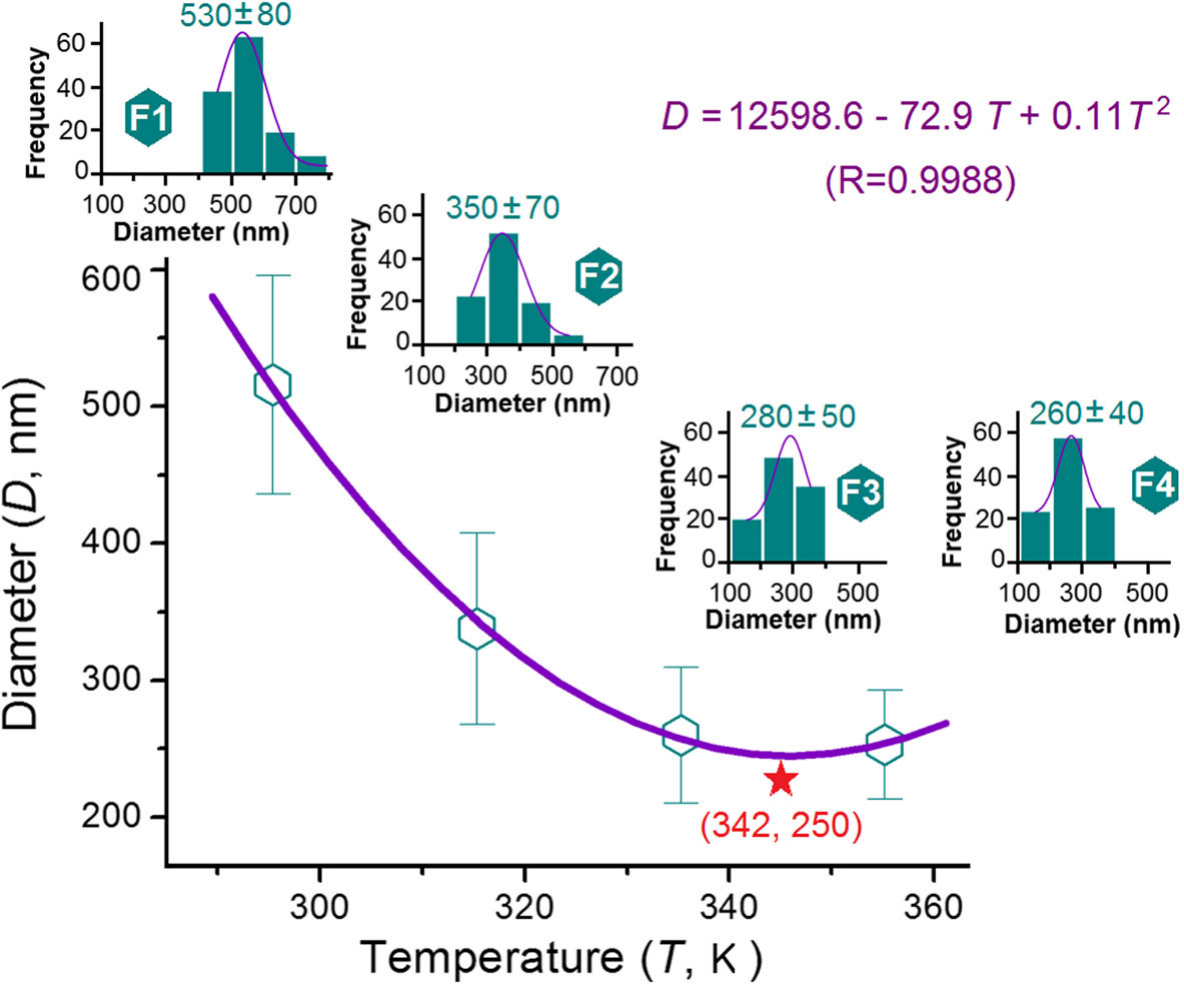
Relationships between the working temperature and the diameters of resultant PAN nanofibers. The *upper-left* to *lower-right insets* show the statistical results of PAN nanofibers F1, F2, F3, and F4

The statistical results of all four PAN nanofibers are shown in Fig. 4. The PAN nanofibers became gradually smaller as the working temperature was increased from 20 to 60 °C. The corresponding PAN nanofibers F1, F2, and F3 featured average diameters of 530 ± 80, 350 ± 70, and 280 ± 50 nm, respectively. However, when the working temperature was further elevated to 80 °C, the resultant PAN nanofibers possessed an average diameter of 260 ± 40 nm, suggesting that the formation of PAN nanofibers was not influenced by the further increase in temperature from 60 to 80 °C.

The data were fit according to different traditional equations, including linear equation (*y* = a*x* + b), power function equation (*y* = a*x* ^b^), and one-variable quadratic equation (*y* = a*x* ^2^ + b*x* + c). Among these equations, the quadratic equation *D* = 12598.6 − 72.9*T* + 0.11*T* ^2^ showed the best fitting result, with a correlation coefficient of 0.9988 (Fig. 4). In the above equation, *D* represents the average diameter of nanofibers (nm) and *T* represents the working temperature (K). According to this equation, an inflection point can be found at *T* = 342 K, i.e., 69 °C. Under this working temperature, the thinnest PAN nanofiber with a diameter of 250 nm was achieved (as indicated by the red star in Fig. 4).

Wang et al. [16] produced PAN fibers with a diameter of 65 nm–85 nm from a 6% PAN solution (no information about molecular weight) at a working temperature of 88.7 °C. A scaling law of *d* = 3.0*η* ^0.74^ (*d* is the fiber diameter and *η* is the viscosity at the working temperature) was deduced. Thus, they concluded that high temperature induces the production of ultrathin fibers. According to their equation, higher working temperature indicates smaller *η* of the working fluid and, thus, smaller diameter of resultant nanofibers. The fundamental explanation that supports this result is that an increase in temperature would decrease the viscosity of polymer solutions but exert minimal influence on physical entanglements, which act to prevent capillary breakup for the formation of linear electrospun nanofibers from polymer solutions. Evidently, our results do not agree with this declaration.

**Fig. 5:**
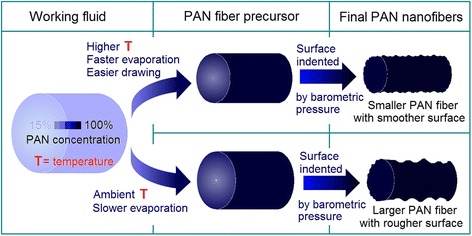
Diagram of the influences of working temperature on fiber formations, as reflected mainly in the surface morphologies and diameters of the resultant nanofibers

A schematic of the influence of working temperature on fiber formation is suggested in Fig. 5. The as-prepared PAN nanofibers significantly differed in fiber diameter and surface roughness depending on the temperature elevation. An elevated working temperature exerts two contradictory effects on the size of resultant nanofibers. During electrospinning with a high temperature, the viscosity and surface tension of polymer solutions decrease, whereas their conductivities decrease [16]. These factors contribute to the effective drawing of electrical forces to generate fibers with small diameters. However, a high temperature could directly influence the electrospinning processes by accelerating the solvent evaporation while indirectly influencing the physicochemical properties of working fluids, which is neglected previously. Ideally, the elongation and thinning of fluid jets should be continuous until the material is deposited on the collector. However, the faster solvent evaporation under high temperatures than under ambient conditions may prematurely stop the electrical stretching processes because of the rapid increase in inner viscoelastic forces of working fluids. PAN fibers were already rigid long before they reached the collector, and the fiber-collected distance did not match the entire electrospinning running processes (from a Taylor cone to a straight fluid jet and to the instable region). Thus, from a solvent evaporation standpoint, the high working temperature was detrimental to the formation of thinner nanofibers. These two factors exert a completing influence, which indicates the presence of an inflection point in Fig. 4.

The faster solvent evaporation under high temperatures than under ambient conditions should create different rigid PAN fiber precursors (which could not be further drawn under the electrical fields) with less content of the residual DMAc. Logically, the later evaporation of the solvent from the native polymeric nanofibers assembled on the collector would deform the final nanofibers, often generating a rough surface owing to barometric pressure [28]. Thus, the final PAN fibers electrospun at high temperatures exhibited smaller surface indentations and a relatively smoother surface morphology compared with those electrospun at relatively low working temperatures (Fig. 5).

### Influence of Temperature on The Formation of PVP Nanofibers

To investigate further the influence of temperature and the related solvent evaporation on the formation of nanofibers, a different solution system consisting of PVP dissolved in anhydrous ethanol (boiling point of 78.4 °C, a protic and volatile solvent, different with DMAc, which is an aprotic solvent with a high boiling point of 166 °C) was explored on the elevated temperature processes. The preparations of PVP nanofibers F5, F6, F7, and F8 at ambient temperature 30, 40, and 50 °C could be implemented continuously and robustly. However, electrospinning at a high temperature of 60 °C for producing PVP nanofiber F9 was fragile and unsteadily. The generation of ethanol vapor from the PVP solution induced the separation of the working fluids into two separate phases in the glass syringe. In the PVP solution, the separated ethanol vapor caused intermittent stoppage of the spinning process and sometimes spurted a pool of liquids.

**Fig. 6:**
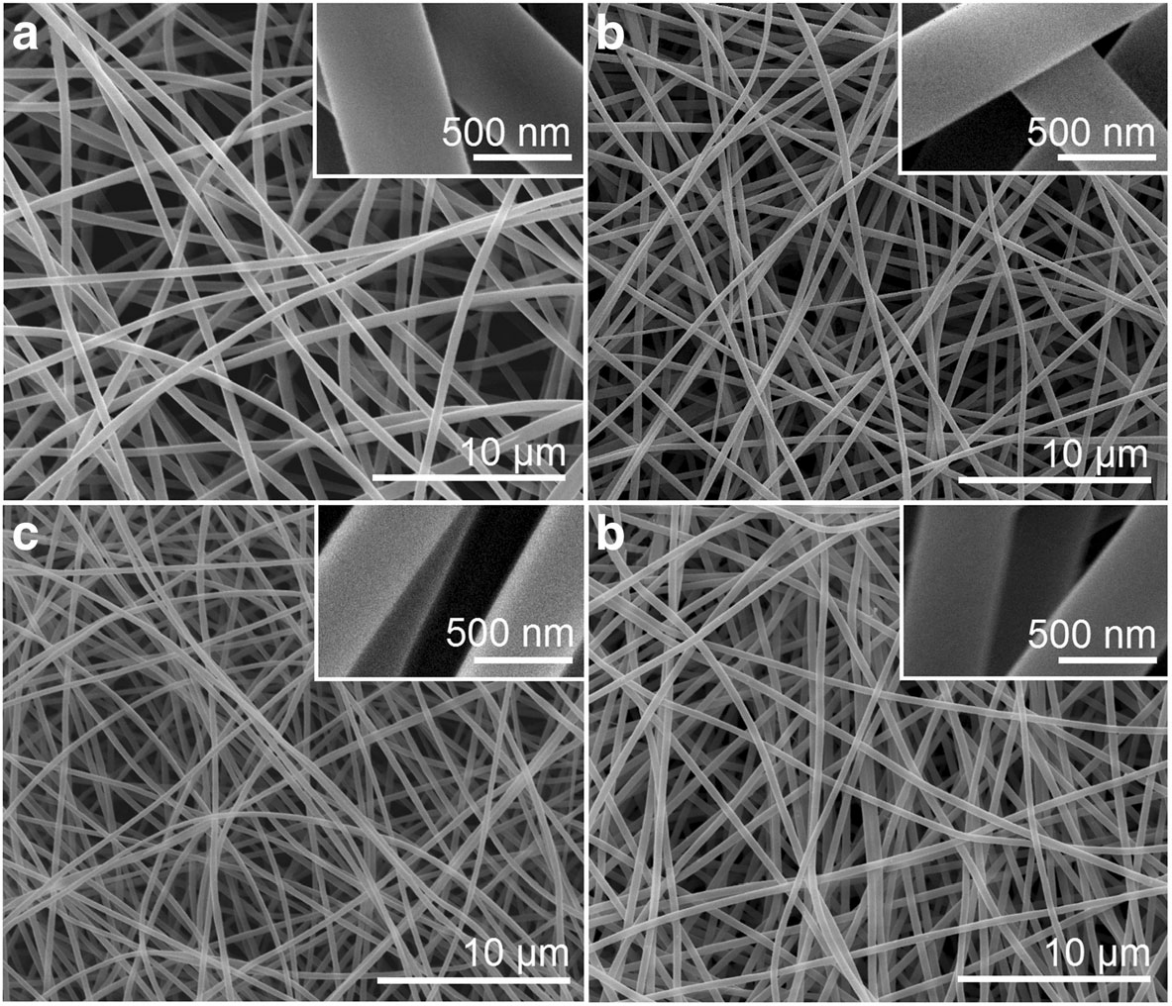
SEM images of the prepared PVP fibers under different working temperatures. **a** F5, ambient temperature (20 °C), **b** F6, 30 °C, **c** F7, 40 °C, and **d** F8, 50 °C. Their *upper-right insets* show enlarged images of the corresponding PVP nanofibers

The prepared PVP nanofibers F5 to F8 exhibited good linear morphology and uniform distributions (Fig. 6). The enlarged SEM images in their upper-right corners demonstrated that all PVP nanofibers possessed a smooth surface without any wrinkles or indentations. The good volatility of ethanol induced the evaporation of all solvents near ethanol from the PVP fluid jets, leaving minimal residual ethanol to escape from the native PVP nanofibers regardless of the varied working temperature. Under these situations, the barometric pressure cannot deform the surfaces of PVP nanofibers.

**Fig. 7:**
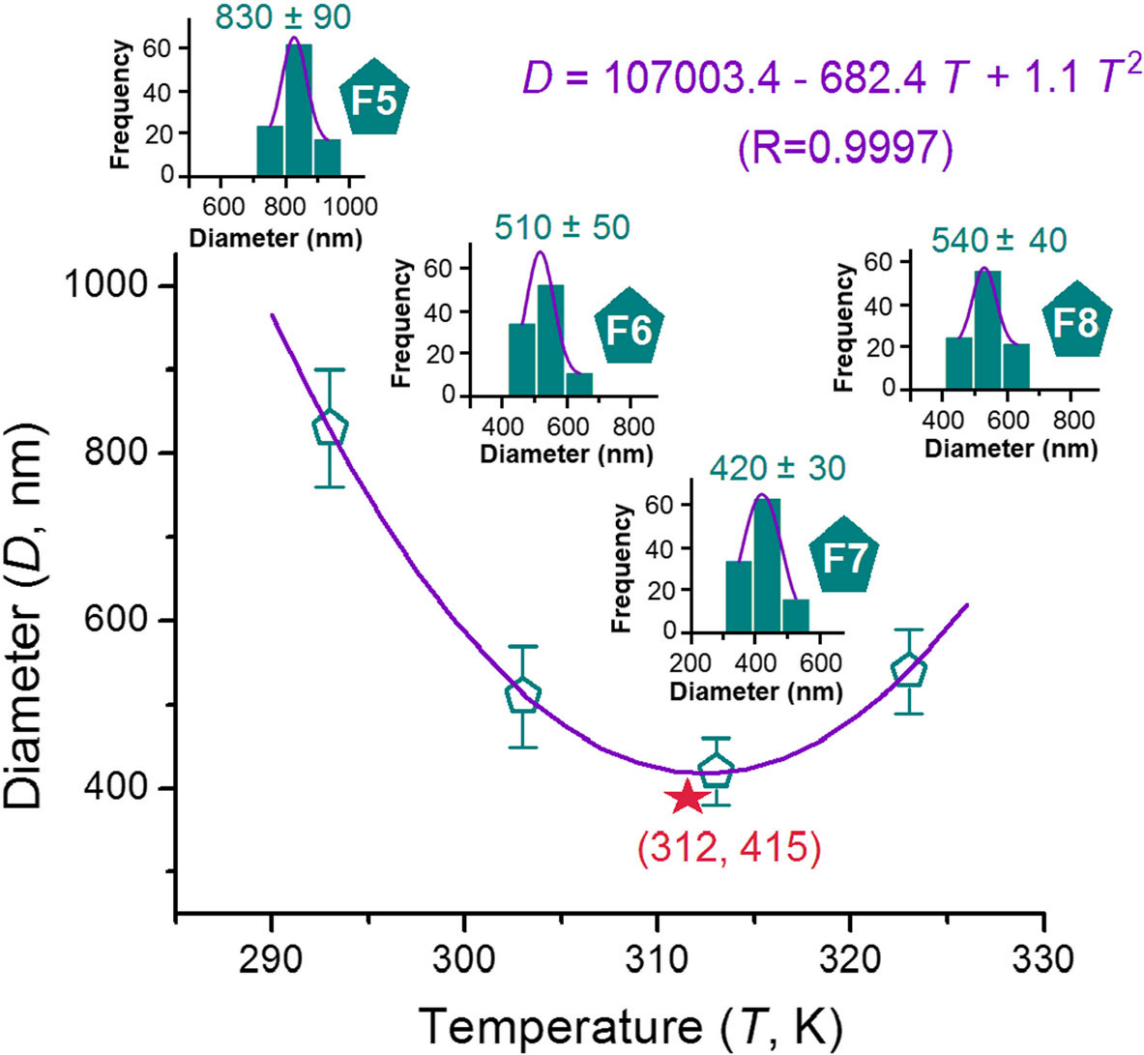
Relationships between the working temperature and the diameters of resultant PVP nanofibers. *Insets* from *left* to *right* show the statistical results of PVP nanofibers F5, F6, F7, and F8

The statistical results of all four PVP nanofibers are shown in Fig. 7. The four PVP nanofibers prepared at 20 to 50 °C working temperature possessed average diameters of 830 ± 90, 510 ± 50, 420 ± 30, and 540 ± 40 nm, showing a clearer trend than the changes in PAN nanofiber diameters with temperature. To fit the data according to the quadratic equation (*y* = *ax* ^2^ + *bx* + *c*), an equation of *D* = 107003.4 − 682.4*T* + 1.1*T* ^2^ with a correlation coefficient of 0.9997 (Fig. 7) was achieved. In this equation, *D* represents the average diameter of nanofibers (nm) and *T* represents the working temperature (K). According to this equation, the inflection point is at *T* = 312 K, i.e., 39 °C. Under this working temperature, the thinnest PVP nanofiber with a diameter of 415 nm was achieved (as indicated by the red star in Fig. 7).

**Fig. 8:**
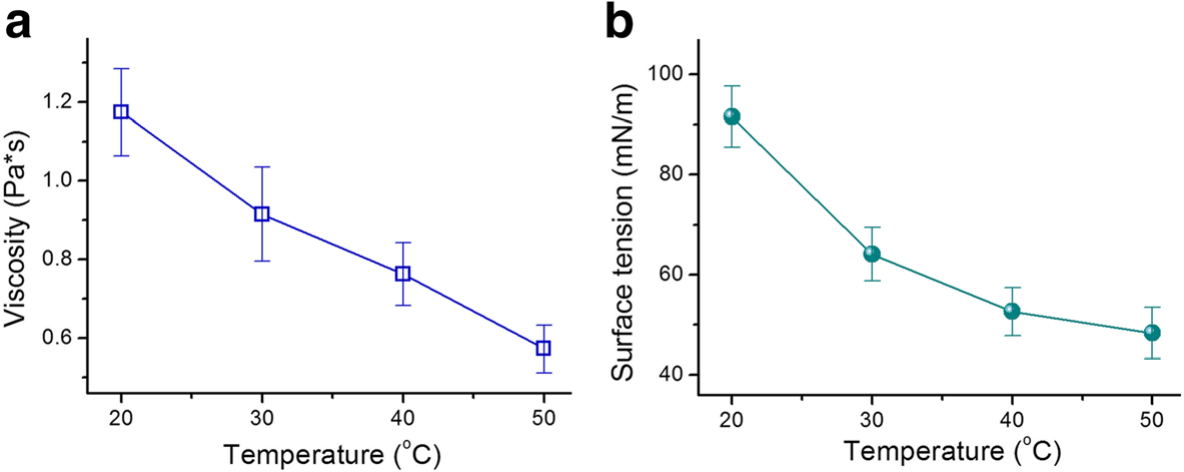
Influence of temperature on the properties of PVP working fluids. **a** Change trend of PVP solution viscosities with temperature. **b** Variation tendency of the surface tension of PVP solutions with temperature

Using electrospinnable PAN solutions, Wang et al. [16] carefully investigated the influence of working temperature on the physicochemical properties, including viscosity, surface tension, and conductivity, of nanofibers. However, ethanol should be a better representative than DMAc because most of the reported polymeric nanofibers in literature were fabricated using solvents with good volatilities. As expected, both the viscosity (Fig. 8a) and surface tension (Fig. 8b) of PVP solutions gradually decreased with the increase in working temperature from 20 to 50 °C. These decreases facilitated easy electrical drawing of PVP fluid jets and the consequent creation of PVP nanofibers with finer diameters. However, their positive effect of reducing nanofibers was counteracted by the rapid solvent evaporation when the temperature increased to the inflection point (i.e., 39 °C). At this point, the rapid solvent evaporation will be a dominant factor in generating nanofibers with increased diameters.

**Fig. 9:**
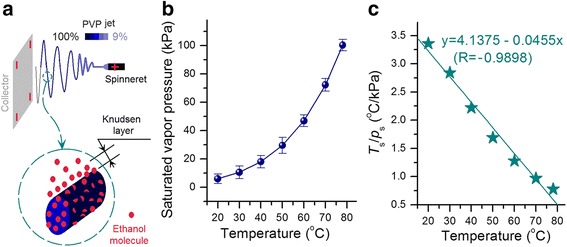
Influence of temperature on the evaporation of ethanol from the fluid jet. **a** Schematic showing the escape of ethanol molecules from the PVP fluid jet through the Knudsen layer. **b** Change in ethanol-saturated vapor pressure with temperature. **c** Change trend of the Knudsen layer thickness with temperature

The evaporation rate of a solvent from a liquid shows a close relationship to the solvent-saturated vapor pressure (which is determined by the surrounding temperature). Near the liquid exists a thin layer of vapor, which is often termed as the evaporation layer or Knudsen layer (Fig. 9a), named after Danish physicist Martin Knudsen. This layer is dynamic and dominates the gas behavior [29]. Thinner Knudsen layer indicates easier escape of ethanol molecules from PVP fluid jets to the atmosphere through this layer and consequently faster solidification of the fluid jets.

The Knudsen layer thickness (*L*_c_) can be estimated according to the following equation [29]:

$$ {L}_{\mathrm{c}}=\frac{k{T}_{\mathrm{s}}}{\pi {d}^2{p}_{\mathrm{s}}} $$

where *p*_s_ is the saturated pressure, *T*_s_ is the temperature, *d* is the solvent molecular diameter, and *k* is the Boltzmann constant.

As evidently seen from the equation, the elevation of temperature will result in a thicker Knudsen layer. However, the increase in temperature will simultaneously increase the saturated pressure and consequently result in a thinner Knudsen layer. As the temperature was gradually increased, *p*_s_ increased faster until the boiling point (Fig. 9b). During temperature increase, the value of *T*_s_/*p*_s_ (which directly reflects the thickness of the Knudsen layer) became smaller and smaller (Fig. 9c). The Knudsen layer thickness presents a virtually linear relationship to temperature, i.e., *y* = 4.1375 − 0.0455*x* (*R* = −0.9898), where *y* is *T*_s_/*p*_s_ and *x* is the temperature. At 60 °C, the thickness of the Knudsen layer was only 38% of that at 20 °C. Thus, higher working temperature indicates smaller *T*_s_/*p*_s_ value and thinner Knudsen layer, corresponding to the faster evaporation of the solvent and solidification of the PVP fluid jets. The premature solidification of the fluid jet under an excessively high temperature will exert a negative influence to stop the drawing under the electric field, thereby producing fibers with large diameters.

## Conclusions

With PAN solutions in DMAc and PVP K90 solutions in ethanol as the model working fluids, elevated temperature electrospinning processes were successfully carried out to synthesize nanofibers under a series of working temperatures by using a homemade electrospinning system. Regardless of protic solvent (ethanol) or aprotic solvent (DMAc) and their volatilization property, elevated working temperature generated both positive and negative influences on the formation of polymer nanofibers. Temperature elevation decreased surface tension and viscosity, which consequently resulted in facile electrospinning and downsized resultant products. However, temperature elevation also directly influenced the electrical drawing during electrospinning by accelerating the evaporation of the solvent from fluid jets. Excessively high working temperatures led to the premature termination of the electrical stretching of polymer fluid jets, thus exerting a negative influence on the produced fibers with large diameters. The PAN and PVP nanofibers produced under reasonable conditions exhibited fine linear morphology without any observed beads-on-a-string or spindles-on-a-string phenomenon. The fitting equations for PAN and PVP nanofibers are *D* = 12598.6 − 72.9*T* + 0.114*T* ^2^ (*R* = 0.9988) and *D* = 107003.4 − 682.4*T* + 1.1*T* ^2^ (*R* = 0.9997), respectively. Given the fact that numerous polymers are sensitive to temperature and numerous functional ingredients exhibit temperature-dependent solubility, the present work serves as a valuable reference for creating novel functional nanoproducts through elevated temperature electrospinning. Manipulating the working temperature can also be combined into the coaxial, side-by-side, and tri-axial electrospinning processes to extend the applications of these techniques in creating novel functional nanomaterials.
